# Spatial distribution of fifty ornamental fish species on coral reefs in the Red Sea and Gulf of Aden

**DOI:** 10.3897/zookeys.367.5476

**Published:** 2014-01-06

**Authors:** Maroof A. Khalaf, Mohamed Abdallah

**Affiliations:** 1 The University of Jordan, Department of Marine Biology, Faculty of Marine Sciences, P. O. Box 195, Aqaba, Jordan; 2 Fishery resources expert, Ministry of Environment, P. O. Box: 8703, Doha, State of Qatar

**Keywords:** Fish community structure, coral reef fishes, Aquarium fishes, Biogeographical affinities, Red Sea and Gulf of Aden

## Abstract

The spatial distribution of 50 ornamental fish species from shallow water habitats on coral reefs were investigated using visual census techniques, between latitudes 11−29°N in the Red Sea, in Jordan, Egypt, Saudi Arabia, and Yemen, and in the adjacent Gulf of Aden in Djibouti. One hundred eighteen transects (each 100×5 m) were examined in 29 sites (3−8 sites per country). A total of 522,523 fish individuals were counted during this survey, with mean abundance of 4428.2 ± 87.26 individual per 500 m² transect. In terms of relative abundance (RA), the most abundant species were Blue green damselfish, *Chromis viridis* (RA=54.4%),followed bySea goldie, *Pseudanthias squamipinnis* (RA= 34.7), Whitetail dascyllus, *Dascyllus aruanus* (RA= 2.6%), *Marginate dascyllus*, *Dascyllus marginatus* (RA= 2.0),Red Sea eightline flasher *Paracheilinus octotaenia* (RA=1.0),andKlunzinger’s wrasse, *Thalassoma rueppellii* (0.7%). The highest number of species (S) per 500 m² transect was found on reefs at the latitude 20° in Saudi Arabia (S=21.8), and the lowest number of species was found at the latitude 15° in Djibouti (S=11.11). The highest mean abundance (8565.8) was found on reefs at latitude 20° in Saudi Arabia and the lowest mean abundance (230) was found on reefs at latitude 22°, also in Saudi Arabia. Whereas, the highest Shannon-Wiener Diversity Index was found in reefs at the latitude 22° (H`=2.4) and the lowest was found in reefs at the latitude 20° (H`=0.6). This study revealed marked differences in the structure of ornamental fish assemblages with latitudinal distribution. The data support the presence of two major biogeographic groups of fishes in the Red Sea and Gulf of Aden: the southern Red Sea and Gulf of Aden group and the group in the northern and central Red Sea. Strong correlations were found between live coral cover and the number of fish species, abundance and Shannon-Wiener Diversity indices, and the strength of these correlations varied among the reefs. A conclusion was done that environmental differences among the reefs and the habitats investigated were important components of abundance variations and species diversity of ornamental fish along latitudinal gradients in the Red Sea and the Gulf of Aden.

## Introduction

Coral reefs are valuable and important ecosystems. They are among the most productive and diverse of all-natural ecosystems, and they are often called «the tropical rain forests of the sea» (Bourilere and Harmelin 1989). Coral reefs have many functions, amongst which is the provision of a variety of habitats for a wealth of organisms. Fishes are a dominant group of coral reef fauna, in terms of both their biomass and diversity. They are the most visible and important mobile component in the coral reefs (Letourneur et al. 1999). Reef ecosystems provide fishes with shelter, feeding, spawning and nursery grounds. Pollution, destructive fishing, over-fishing, coral bleaching, tourism development and other stresses and activities threaten these valuable ecosystems.

While the Red Sea fish fauna is taxonomically quite well known (Randall 1992, Goren and Dor 1994, Khalaf 2004) compared with other parts of the tropical Indo-Pacific Ocean, the community structure of shore fishes has been less well investigated (Khalaf and Kochzius 2002). Despite a long tradition of taxonomical work (e.g. Forsskal 1775, Klunzinger 1884), few studies have been published on the general community structure of the Red Sea shore fishes (Bouchon-Navaro 1980, Ben-Tuvia et al. 1983, Rilov and Benayahu 2000, Khalaf and Kochzius 2002, Alwany and Stachowitsch 2007) and these studies cover a small range of spatial distribution. Most species of near-shore fishes in the Red Sea associate with coral reefs, although some occur in sea grasses, sandy areas, and other near-shore habitats (UNEP 1997). The present study was carried out through the Sustainable Use and Management of Living Marine Resources component of the Strategic Action Programme for the Red Sea and Gulf of Aden (SAP) executed by The Regional Organization for the Conservation of the Environment of the Red Sea and Gulf of Aden (PERSGA) between 1999 and 2003. The study was conducted at coral reef sites to assemble comparative regional data on the distribution of fifty ornamental fish species at latitudinal (11–29°N) spatial scales. The main objectives of the study were to assess the community structure, and biogeographic affinities of Red Sea coral reef fishes that are collected for the aquarium trade.

## Material and methods

### Study area

**Figure 1.**
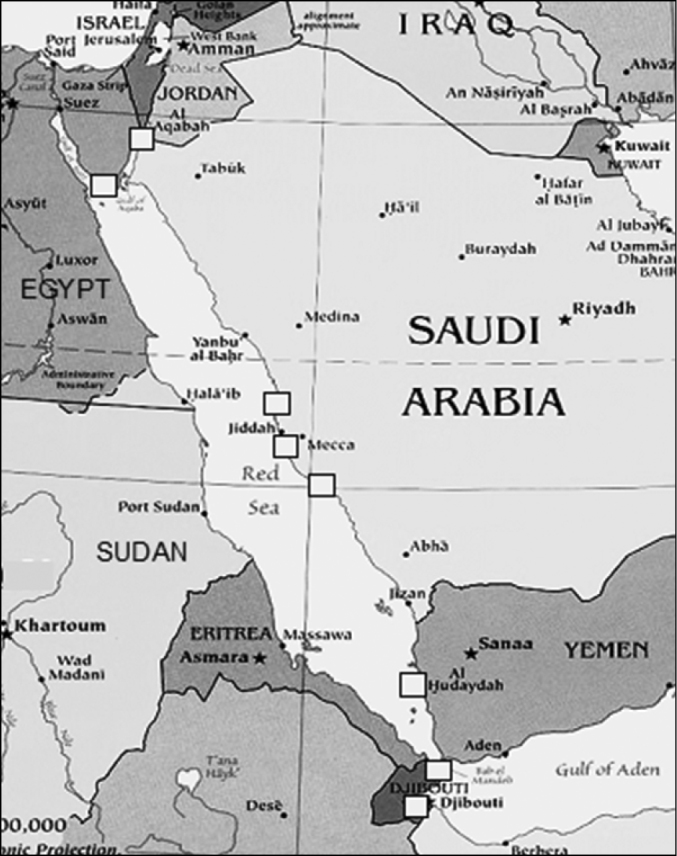
Map of the Red Sea and Gulf of Aden. Squares indicate the coral reef sites examined in the present study.

The study was conducted at coral reef sites at latitudes between 11–29°N along the coasts of Jordan, Egypt, Saudi Arabia, Yemen in the Red Sea and just outside the Red Sea in the Gulf of Aden, for Djibouti (Fig. 1).

### Visual census

We selected 50 ornamental reef fish species that occur in shallow water habitats on coral reefs only. Based on the information collected from fishermen and Government authorities in member countries of (PERSGA), most of the sites and depths in Egypt, Saudi Arabia and Yemen were selected in sites used by collectors of the ornamental fishes. Whereas, the sites in Jordan and Djibouti were collection of ornamental fishes did not take place was carried out in coral reef sites. The abundance of each species was surveyed along the coasts of Jordan, Egypt, Saudi Arabia, Yemen and Djibouti using a visual census technique by SCUBA divers, as described in English et al. (1994). The visual census technique is a widely used method for ecological studies of fishes on coral reefs. However, differences in skill levels and techniques of observers can be a source of imprecision and/or bias (Thompson and Mapstone 1997). Therefore, only underwater fish counting performed by the first author was used to avoid this type of bias in the present study. A total of 118 transects, each 100m length × 5m width (500m²) were performed at 29 sites (3–6 transects per site except at Ras Mohamed two transects instead of three at 2–4 m depth were surveyed due to the strong water currents occurred during the period of study, Table 1). At each site, a visual census was conducted along three transects, in most cases on the shallow reef slope (4–5 m) and also three transects on the deep reef slope (8–10m). Divers then swam along the transects and recorded individuals of the 50 selected fish species within 2.5 m either side, and 3 m above the transect line for a duration of 25–30 minutes. Fish identification in the field was checked using published guides (Myers 1991, Randall 1992, Smith and Heemstra 1986, Khalaf and Disi 1997). The common name of fishes indicated in this study were after Froese and Pauly 2000 (FishBase 2013). All latitude numbers are for north latitudes.

**Table 1.** Information on 29 sampling sites along the Jordanian, Egyptian, Saudi Arabian, Yemeni and Djibouti coasts, Red Sea and Gulf of Aden; n=number of transects examined; date of fish counts given for each sampling depth, and position of each site in latitude N and longitude E shown. NR=not recorded. Note: in Yemen, Djibouti, and at some sites in Saudi Arabia, only transects at 2−4m depth were examined.

**Country**	**Latitude**	**Site**	**n**	**2–4m**	**n**	**5 m depth**	**n**	**10 m depth**	**N**	**E**	**Total No. of transects**
Jordan	JO 29°	Marine Science Station			3	26/7/2002	3	26/7/2002	29°26.276, 34°58.275		
		Visiting Center			3	25/7/2002	3	25/7/2002	NR	NR	18
		Tourist Camp			3	24/7/2002	3	24/7/2002	29°26.276, 34°58.275		
Egypt	EG 27°	Noksh			3	11/7/2002	3	11/7/2002	27°46.723, 34°02.915		
		Mahmoudat			3	12/7/2002		12/7/2002	27°44.460, 34°08.881		
		Manar Dolphin			3	13/7/2002	3	13/7/2002	27°42.303, 34°07.133		31
		Elli			3	14/7/2002	3	14/7/2002	27°47.334, 33°53.227		
		Zorab			5	16/7/2002		16/7/2002	27°50.146, 34°00.230		
		Ras Mohamad			2	15/7/2002	3	15/7/2002	27°43.886, 34°15.663		
Saudi Arabia	SA 22°	Bostek/Thoal					3	2/11/2002	22°19.647, 39°02.350		6
		Thoal- Awjam			3	2/11/02			22°19.648, 39°02.351		
	SA 21°	Alkabeera			3	30/10/2002	3	27/10/2002	21°41.581, 39°00.741		21
		Bayada			3	28/10/2002			NR	NR	
		South Batch Bayada			3	30/10/2002	3	29/10/2002	21°44.602, 38°57.798		
		Al-Sagheera			3	31/10/2002			21°39.721, 38°58.850		
		Al-Kherq			3	31/10/2002			21°43.039, 38°59.253		
	SA 20°	Alleeth			3	3/11/2002	3	3/11/2002	20°06.326, 40°13.030		6
Yemen	YE 15°	Kadaman	3	8/10/2002					15°33.949, 42°13.585		18
		Kamaran	3	5/10/2002					15°16.593, 42°36.192		
		Tekfash	3	7/10/2002					15°41.979, 42°23.654		
		Quish	3	7/10/2002					15°41.084, 42°28.110		
		Al-murk	3	8/10/2002					NR	NR	
		Al-Badi	3	9/10/2002					NR	NR	
Djibouti	DJ 12°	Gehere	3	17/10/2002					12°16.662, 43°22.926		3
	DJ 11°	Khor Ambado	3	13/10/2002					11°35.780, 43°01.985		15
		Maskali	3	14/10/2002					11°42.937, 43°09.246		
		Musha	3	14/10/2002					11°44.669, 43°12.440		
		Tajoura	3	15/10/2002					11°46.245, 42°56.860		
		Arta Plaga	3	16/10/2002					11°35.394, 42°49.981		
		Total n	36		49		33				118

Surveys of the benthic habitat were carried out by assistants from Saudi Arabia and Yemen. The line-point intercept method was used with the tape stretched taut 200 points recorded per 100-m transect. From this point-intercept data, the percentage cover was calculated of live hard coral, live soft coral, dead coral, coral rock, sand, rubble, macro algae, algal turf, sponge, and others as described by Edwards (2002).

### Statistical analysis

Abundance of fishes was described by relative abundance (RA) and frequency of appearance (FA) following Khalaf et al. (2006). RA was calculated as: (pooled average abundance of species *i* at each depth and site / pooled average abundance of all species at each depth and site) × 100. FA was determined as: (number of transects in which species *i* was present / total number of transects) X 100. Species richness (number of species), and Shannon-Weiner species diversity were calculated using PRIMER-E (Polymouth Marine Laboratory, UK, 2000). Multivariate analysis of the data such as cluster analysis was performed using the same software. Analysis of a linear regression test used to examine the relationship between hard coral (HC) and soft coral (SC) vs. species richness (S), fish abundance (N), Shannon-Weiner Diversity Index (H´) as described by Sokal and Rohlf (1981) and implemented using STAT VEIW computer software.

## Results

### Benthic habitat

**Figure 2.**
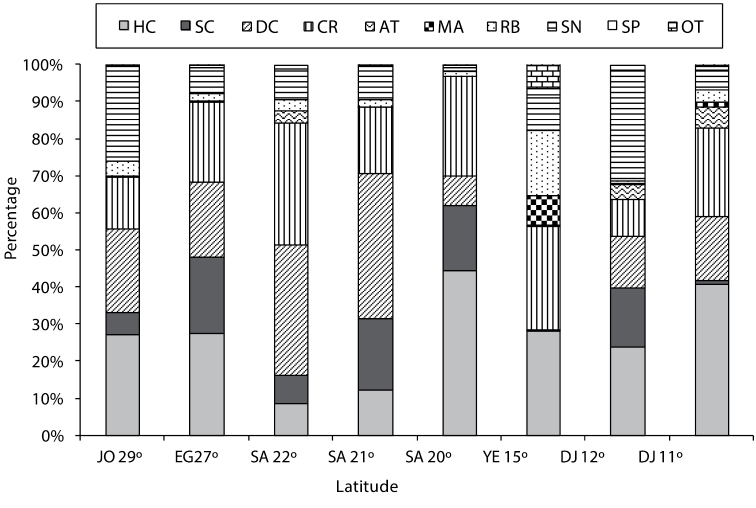
Percent cover (%) for substrate types at all latitudes [(JO=Jordan, EG=Egypt, SA= Saudi Arabia, YE=Yemen, DJ=Djibouti), where (HC=Hard coral, SC=Soft coral, DC=Dead coral, CR=Coral rock, AT=Algal turf, MA=Macroalgae, RB=Rubble, SN=Sand, SP=Sponge, OT=Others)].

Fig. 2 shows the percentage cover of substrate types for various latitudes along the Red Sea and Gulf of Aden.

**Jordan**

Latitude 29°

Along the Jordanian Gulf of Aqaba coast, the highest hard live coral cover was 47%, occurring at the Visitor Center at 10 m depth. The average live hard coral cover across all sites in Jordan was 27%. The highest soft coral cover was at the Visitor Center at 5 m depth, and the average among all Jordanian sites was 6%.

**Egypt**

Latitude 27°

Along the Egyptian coast, the highest hard coral cover was 41.2%, found at both Noksh (5 m depth) and at Mahmoudat (5 m depth). The average of hard coral cover at all Egyptian sites was 27.6%. The highest soft coral cover was 47.8% at Fanar Dolphin and Ras Mohammed respectively, both at 10 m depth. The average soft coral cover at all sites in Egypt was 20.6%.

**Saudi Arabia**

Latitude 22°

At latitude 22°, the highest hard coral cover was 14%, occurring at Thowal-Awjam at 4 m depth. The average hard live coral at all sites at this latitude was 8.8%. The highest soft coral cover was 5.5% in Thoal-Bostek, at 10 m deep, and the average soft coral cover at all sites at this latitude was 7.7%.

Latitude 21°

The highest hard live coral cover was 35.5% at Bayada, in the deep transect. The average hard live coral cover at all sites was 12.4%. The highest soft coral cover was 81.5% at Alkabeera at 4m deep, and the average soft coral cover at all sites was 19.2%.

Latitude 20°

The highest hard coral cover was 52.5% at Alleeth in shallow transects. The average hard live hard coral in all transects was 44.4%. The highest soft coral cover was 32.0%, and the average of all transects was 17.5%.

**Yemen**

Latitude 15°

The highest hard coral cover along the Yemeni Red Sea Coast was 40.5% at Tekfash. The average live hard coral cover at all sites was 26.2%. The highest soft coral was 0.3% at Tekfash. The average soft coral cover at all sites was 0.1%.

**Djibouti**

The below sites were in the Gulf of Aden.

Latitude 12°

The highest hard coral cover was 32.5% recorded at Gehere in shallow transects. The average hard live coral cover at all sites was 23.8%. The highest soft coral cover was 17.5% recorded at Gehere in shallow transects. The average of soft coral cover at all sites was 15.8%.

Latitude 11°

The highest live hard coral cover was 71.0% at Tadjoura in shallow transects. The lowest hard live coral cover was 14.0% at Maskali in shallow transects. The highest soft coral cover was 2.5% at Musha. The average soft coral cover at all transects was 3.4%.

The overall mean percentage cover for both hard and soft coral (HC and SC combined) from highest to lowest was as follows: The highest was 61.9% total coral cover at latitude 20° along the Saudi Arabia coast, then 48.2% at latitude 27° along the Egyptian coast, 44.1 and 39.7 at latitude 11 and 12 ° respectively, 33.3% at latitude 29° along the Jordanian coast, 31.6 at Latitude 21 at Saudi Arabia coast, 26.3% at latitude 15° at the Yemeni coast, and the lowest combined total coral cover was 16.4% at latitude 22° along the Saudi Arabia coast.

### Fish assemblages and community indices. Dominant taxa and fish community structure

**Jordan**

Latitude 29°

**Figure 3.**
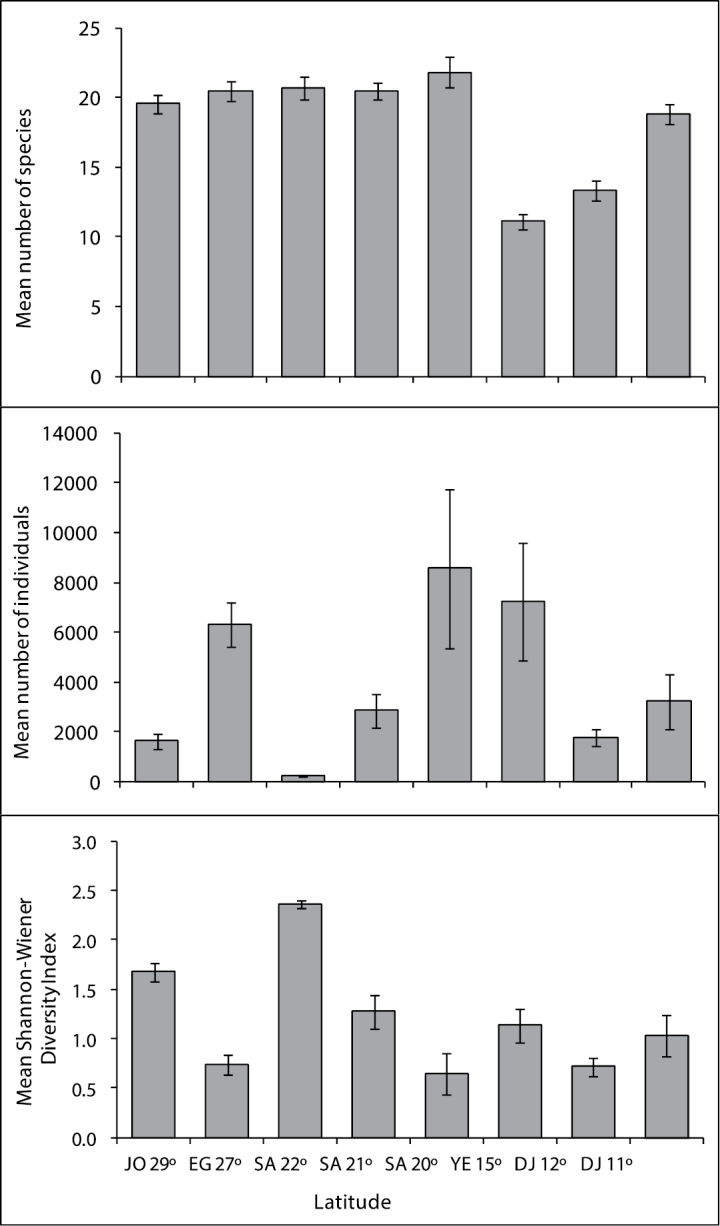
Mean number of fish species, mean number of individuals, and mean Shannon-Wiener Diversity Index in coral reef along the Jordan latitude 29°; Egypt latitude 27°; Saudi Arabia latitude 22, 21, 20°; Yemen 15° and Djibouti latitude 12, 11°.

A total of 29,485 fishes were counted along the 18 transects examined at latitude 29°. The mean fish abundance ranged from 475 individuals per transect on the Tourist Camp reef at 5 m depth, to 3,117 individuals per transect on the Visitor Center reef at 5 m depth. The mean fish abundance across all transects was 1,638 individuals per transect. Of the 50 fish species considered, only 35 were observed at Jordanian sites. The number of species observed ranged from 23 at 10m depth at the Tourist Camp, to 30 at 5m depth at the Marine Science Station, with an average of 19.6 species per transect (Fig. 3). In terms of relative abundance (RA) on Jordanian reefs, the 5 most abundant fish species examined was the Sea goldie, *Pseudanthias squamipinnis* (Peters, 1855) (RA= 40.7%), followed by Blue green damselfish, *Chromis viridis* (Cuvier, 1830)(RA= 27.5%), Whitetail dascyllus, *Dascyllus aruanus* (Linnaeus, 1758) (RA= 7.7%), Red Sea eightline flasher, *Paracheilinus octotaenia* Fourmanoir, 1955 in Roux-Estève and Fourmanoir 1955 (RA=7.3%), and Marginate dascyllus, *Dascyllus marginatus* (Rüppell, 1829) (RA= 6.8%). Together, these five species made up 90% of the abundance of the 50 fish species examined (Table 2).

**Table 2.** Relative abundance in % of fish individuals per 500m² transect along reefs at JO= Jordan latitude 29°; EG=Egypt latitude 27°; SA=Saudi Arabia latitude 22, 21, 20°; YE=Yemen 15° and DJ=Djibouti latitude 12, 11°. (TRA indicates total relative abundance per family).

**Fish scientific name**	**Fish common name**	**JO 29°**	**EG 27°**	**SA 22°**	**SA 21°**	**SA 20°**	**YE 15°**	**DJ 12°**	**DJ 11°**	**Total RA**
**Scorpaenidae**										0,01
*Pterois miles*	Devil firefish	0,0	0,0	0,0	0,0	0,0	0,0	0,0	0,0	0,00
*Pterois radiata*	Radial firefish	0,0	0,0	0,0	0,0	0,0	0,0	0,0	0,0	0,01
**Serranidae**										34,71
*Pseudanthias squamipinnis*	Sea goldie	40,7	84,6	16,7	3,9	1,5	0,0	0,0	0,0	34,71
**Pseudochromidae**										0,28
*Pseudochromis flavivertex*	Sunrise dottyback	0,0	0,0	6,7	0,0	0,0	0,0	0,0	0,0	0,03
*Pseudochromis fridmani*	Orchid dottyback	0,8	0,4	0,9	0,2	0,1	0,0	0,0	0,0	0,23
*Pseudochromis springeri*	Blue-striped dottyback	0,1	0,0	0,0	0,0	0,0	0,0	0,0	0,0	0,02
**Chaetodontidae**										0,96
*Chaetodon auriga*	Threadfin butterflyfish	0,0	0,2	1,2	0,1	0,0	0,0	0,0	0,0	0,10
*Chaetodon austriacus*	Blacktail butterflyfish	0,5	0,2	2,3	0,2	0,0	0,0	0,0	0,0	0,13
*Chaetodon fasciatus*	Diagonal butterflyfish	0,1	0,1	1,3	0,1	0,0	0,0	0,0	0,1	0,06
*Chaetodon larvatus*	Hooded butterflyfish	0,0	0,0	1,2	0,1	0,2	0,8	0,2	0,4	0,27
*Chaetodon lineolatus*	Lined butterflyfish	0,0	0,0	0,1	0,0	0,0	0,0	0,0	0,0	0,00
*Chaetodon melannotus*	Blackback butterflyfish	0,0	0,0	0,0	0,1	0,0	0,0	0,0	0,0	0,03
*Chaetodon melapterus*	Arabian butterflyfish	0,0	0,0	0,0	0,0	0,0	0,0	0,4	0,2	0,02
*Chaetodon mesoleucos*	White-face butterflyfish	0,0	0,0	0,0	0,0	0,0	0,0	0,0	0,1	0,01
*Chaetodon paucifasciatus*	Eritrean butterflyfish	1,5	0,1	0,0	0,1	0,0	0,0	0,0	0,0	0,14
*Chaetodon semilarvatus*	Bluecheek butterflyfish	0,0	0,0	0,1	0,0	0,0	0,0	0,1	0,1	0,03
*Chaetodon trifascialis*	Chevron butterflyfish	0,0	0,1	0,0	0,0	0,0	0,0	0,1	0,1	0,07
*Chaetodon vagabundus*	Vagabond butterflyfish	0,0	0,0	0,0	0,0	0,0	0,0	0,3	0,1	0,01
*Heniochus acuminatus*	Pennant coralfish	0,0	0,0	0,0	0,0	0,0	0,0	0,0	0,0	0,00
*Heniochus intermedius*	Red Sea bannerfish	0,1	0,0	3,4	0,1	0,0	0,1	0,1	0,3	0,10
**Pomacanthidae**										0,18
*Pomacanthus asfur*	Arabian angelfish	0,0	0,0	1,7	0,0	0,0	0,1	0,1	0,1	0,06
*Pomacanthus imperator*	Emperor angelfish	0,0	0,0	0,0	0,0	0,0	0,0	0,0	0,0	0,00
*Pomacanthus maculosus*	Yellowbar angelfish	0,0	0,0	0,5	0,0	0,0	0,3	0,1	0,1	0,08
*Pygoplites diacanthus*	Regal angelfish	0,0	0,0	0,7	0,1	0,0	0,0	0,0	0,1	0,04
**Pomacentridae**										59,39
*Amphiprion bicinctus*	Twoband anemonefish	1,5	0,0	0,4	0,0	0,0	0,0	0,0	0,0	0,12
*Chromis viridis*	Blue green damselfish	27,5	10,4	0,0	73,9	92,3	88,8	83,8	90,8	54,40
*Dascyllus aruanus*	Whitetail dascyllus	7,7	0,2	0,6	17,3	0,1	0,3	0,0	0,4	2,61
*Dascyllus marginatus*	Marginate dascyllus	6,8	0,3	0,0	0,0	0,1	5,8	0,0	0,1	1,95
*Dascyllus trimaculatus*	Threespot dascyllus	0,4	0,0	0,0	0,1	0,1	1,0	0,0	0,0	0,30
**Cirrhitidae**										
*Paracirrhites forsteri*	Blackside hawkfish	0,0	0,1	0,3	0,3	0,0	0,0	0,0	0,0	0,08
**Labridae**										3,45
*Anampses twistii*	Yellowbreasted wrasse	0,6	0,1	0,5	0,1	0,0	0,0	0,0	0,0	0,09
*Bodianus anthioides*	Lyretail hogfish	0,1	0,0	0,4	0,0	0,0	0,0	0,0	0,0	0,02
*Cheilinus lunulatus*	Broomtail wrasse	0,0	0,0	1,3	0,0	0,0	0,0	0,0	0,0	0,04
*Coris aygula*	Clown coris	0,0	0,1	0,1	0,0	0,0	0,0	0,0	0,0	0,03
*Gomphosus caeruleus*	Green birdmouth wrasse	0,5	0,2	6,9	0,6	0,2	0,0	1,2	0,5	0,26
*Labroides dimidiatus*	Bluestreak cleaner wrasse	0,1	0,2	3,6	0,2	0,0	0,0	0,2	0,1	0,12
*Larabicus quadrilineatus*	Fourline wrasse	0,4	0,1	12,3	0,2	0,4	1,6	4,1	1,7	0,74
*Novaculichthys taeniourus*	Rockmover wrasse	0,0	0,0	0,0	0,0	0,0	0,0	0,0	0,0	0,00
*Paracheilinus octotaenia*	Red Sea eightline flasher	7,3	0,3	1,1	0,0	3,9	0,1	0,0	0,7	0,98
*Thalassoma rueppellii*	Klunzinger’s wrasse	2,6	1,2	6,1	0,7	0,2	0,0	0,0	0,0	0,72
*Thalassoma lunare*	Moon wrasse	0,0	0,0	5,6	0,2	0,2	0,6	7,6	1,5	0,45
**Acanthuridae**										0,88
*Acanthurus sohal*	Sohal surgeonfish	0,0	0,7	12,4	0,3	0,3	0,2	0,4	0,5	0,43
*Naso lituratus*	Orangespine unicornfish	0,0	0,1	2,9	0,1	0,1	0,0	0,0	0,8	0,14
*Zebrasoma veliferum*	Sailfin tang	0,1	0,1	6,4	0,5	0,0	0,0	0,0	0,2	0,14
*Zebrasoma xanthurum*	Yellowtail tang	0,3	0,1	0,9	0,2	0,0	0,0	1,2	1,0	0,17
**Balistidae**										0,04
*Balistapus undulatus*	Orange-lined triggerfish	0,0	0,0	0,8	0,1	0,0	0,0	0,0	0,0	0,03
*Balistoides viridescens*	Titan triggerfish	0,0	0,0	0,1	0,0	0,0	0,0	0,0	0,0	0,00
*Rhinecanthus assasi*	Picasso triggerfish	0,0	0,0	0,0	0,0	0,0	0,0	0,0	0,0	0,01
**Ostraciidae**										0,00
*OstraciOstracion cubicus*	Yellow boxfish	0,0	0,0	0,2	0,0	0,0	0,0	0,0	0,0	0,00
**Tetraodontidae**										0,01
*Arothron diadematus*	Masked puffer	0,0	0,0	0,4	0,0	0,0	0,0	0,0	0,0	0,01

In terms of frequency of appearance, the most common species were *Pseudanthias squamipinnis*, *Dascyllus aruanus*, *Dascyllus marginatus*, Twoband anemonefish, *Amphiprion bicinctus* Rüppell, 1830, Eritrean butterflyfish *Chaetodon paucifasciatus* Ahl, 1923 (100% each), *Chromis viridis*, Fourline wrasse, *Larabicus quadrilineatus* (Rüppell, 1835), Green birdmouth wrasse, *Gomphosus caeruleus* Lacepède, 1801, and Yellowbreasted wrasse, *Anampses twistii* Bleeker, 1856 (94.4% each) (Table 3).

**Table 3.** Frequency of appearance in % of fish individuals per 500m² transect along reefs at JO= Jordan latitude 29°; EG=Egypt latitude 27°; SA=Saudi Arabia latitude 22, 21, 20°; YE=Yemen 15° and DJ=Djibouti latitude 12, 11°.

**Fish species**	**JO 29°**	**EG 27°**	**SA 22°**	**SA 21°**	**SA 20°**	**YE 15°**	**DJ 12°**	**DJ 11°**
**Scorpaenidae**								
*Pterois miles*	16,7	29,0	0,0	0,0	0,0	0,0	0,0	0,0
*Pterois radiata*	22,2	29,0	0,0	0,0	16,7	0,0	0,0	20,0
**Serranidae**								
*Pseudanthias squamipinnis*	100,0	80,6	50,0	33,3	83,3	0,0	0,0	0,0
**Pseudochromidae**								
*Pseudochromis flavivertex*	0,0	0,0	50,0	19,0	0,0	22,2	0,0	0,0
*Pseudochromis fridmani*	88,9	74,2	50,0	47,6	33,3	0,0	0,0	0,0
*Pseudochromis springeri*	61,1	25,8	0,0	0,0	0,0	0,0	0,0	0,0
**Chaetodontidae**								
*Chaetodon auriga*	11,1	87,1	83,3	66,7	33,3	0,0	0,0	0,0
*Chaetodon austriacus*	83,3	90,3	100,0	95,2	33,3	0,0	0,0	0,0
*Chaetodon fasciatus*	72,2	74,2	66,7	90,5	50,0	16,7	33,3	66,7
*Chaetodon larvatus*	0,0	3,2	100,0	76,2	100,0	100,0	100,0	100,0
*Chaetodon lineolatus*	0,0	9,7	16,7	4,8	0,0	0,0	0,0	0,0
*Chaetodon melannotus*	27,8	41,9	0,0	33,3	0,0	0,0	0,0	13,3
*Chaetodon melapterus*	0,0	0,0	0,0	0,0	0,0	0,0	100,0	93,3
*Chaetodon mesoleucos*	0,0	0,0	0,0	23,8	16,7	22,2	0,0	66,7
*Chaetodon paucifasciatus*	100,0	80,6	0,0	57,1	16,7	0,0	0,0	0,0
*Chaetodon semilarvatus*	0,0	45,2	16,7	33,3	50,0	50,0	33,3	73,3
*Chaetodon trifascialis*	11,1	74,2	0,0	52,4	0,0	5,6	33,3	33,3
*Chaetodon vagabundus*	0,0	0,0	0,0	0,0	0,0	0,0	100,0	73,3
*Heniochus acuminatus*	0,0	0,0	0,0	0,0	0,0	0,0	0,0	6,7
*Heniochus intermedius*	44,4	67,7	100,0	14,3	100,0	83,3	100,0	93,3
**Pomacanthidae**								
*Pomacanthus asfur*	0,0	0,0	66,7	0,0	83,3	94,4	33,3	86,7
*Pomacanthus imperator*	5,6	19,4	0,0	0,0	16,7	5,6	33,3	0,0
*Pomacanthus maculosus*	0,0	22,6	66,7	0,0	83,3	100,0	100,0	80,0
*Pygoplites diacanthus*	5,6	54,8	66,7	14,3	100,0	0,0	0,0	80,0
**Pomacentridae**								
*Amphiprion bicinctus*	100,0	45,2	33,3	47,6	83,3	11,1	0,0	26,7
*Chromis viridis*	94,4	54,8	0,0	71,4	100,0	55,6	100,0	93,3
*Dascyllus aruanus*	100,0	22,6	33,3	61,9	66,7	16,7	0,0	46,7
*Dascyllus marginatus*	100,0	19,4	0,0	0,0	33,3	50,0	0,0	20,0
*Dascyllus trimaculatus*	44,4	16,1	0,0	4,8	33,3	11,1	0,0	13,3
**Cirrhitidae**								
*Paracirrhites forsteri*	0,0	83,9	33,3	90,5	16,7	0,0	0,0	0,0
**Labridae**								
*Anampses twistii*	94,4	77,4	33,3	66,7	50,0	0,0	0,0	53,3
*Bodianus anthioides*	61,1	29,0	50,0	14,3	16,7	0,0	0,0	0,0
*Cheilinus lunulatus*	22,2	64,5	83,3	14,3	66,7	72,2	33,3	53,3
*Coris aygula*	27,8	67,7	16,7	14,3	0,0	5,6	0,0	0,0
*Gomphosus caeruleus*	94,4	87,1	100,0	100,0	100,0	22,2	100,0	93,3
*Labroides dimidiatus*	66,7	93,5	100,0	100,0	100,0	27,8	100,0	66,7
*Larabicus quadrilineatus*	94,4	74,2	100,0	76,2	100,0	100,0	100,0	100,0
*Novaculichthys taeniourus*	0,0	0,0	0,0	4,8	16,7	0,0	0,0	0,0
*Paracheilinus octotaenia*	55,6	9,7	33,3	0,0	83,3	5,6	0,0	33,3
*Thalassoma rueppellii*	100,0	90,3	83,3	85,7	83,3	0,0	0,0	0,0
*Thalassoma lunare*	22,2	48,4	100,0	81,0	83,3	100,0	100,0	100,0
**Acanthuridae**								
*Acanthurus sohal*	0,0	32,3	66,7	71,4	100,0	50,0	66,7	53,3
*Naso lituratus*	5,6	41,9	50,0	71,4	50,0	5,6	0,0	6,7
*Zebrasoma veliferum*	44,4	48,4	100,0	47,6	83,3	44,4	0,0	86,7
*Zebrasoma xanthurum*	88,9	41,9	33,3	47,6	16,7	22,2	66,7	100,0
**Balistidae**								
*Balistapus undulatus*	50,0	29,0	83,3	76,2	50,0	0,0	0,0	40,0
*Balistoides viridescens*	0,0	3,2	33,3	14,3	16,7	0,0	0,0	13,3
*Rhinecanthus assasi*	0,0	19,4	0,0	42,9	16,7	5,6	0,0	0,0
**Ostraciidae**								
*OstraciOstracion cubicus*	27,8	3,2	16,7	4,8	0,0	5,6	0,0	0,0
**Tetraodontidae**								
*Arothron diadematus*	11,1	32,3	66,7	19,0	16,7	0,0	0,0	0,0

**Egypt**

Latitude 27°

A total of 196,379 fish individuals were counted along 32 transects which were examined at the latitude 27°. The mean abundance ranged from 310 individuals at Mahmoudat at 10 m depth, to 13,358 individuals per transect at Fanar Al-Dolphin at 5m depth. The mean fish abundance of all transects was 6,334 individuals per transect. Of the 50 fish species considered, only 43 were reported from Egyptian sites. The number of species observed ranged from 26 at Mahmoudat (10 m depth) to 34 at Al-Noksh (9 m depth), with a mean of 20.6 species per transect (Fig. 3).

In terms of relative abundance (RA) on Egyptian reefs, the most abundant species was *Pseudanthias squamipinnis* (RA= 84.6%), followed by *Chromis viridis* (RA= 10.4%); these two species made up 95% of all the fishes recorded in Egypt (Table 2).

In terms of frequency of appearance (FA), the most common species was, Bluestreak cleaner wrasse *Labroides dimidiatus* (Valenciennes, 1839 in Cuvier and Valenciennes 1839) at 94.%, followed by Blacktail butterflyfish, *Chaetodon austriacus* Rüppell, 1836 and Klunzinger’s wrasse, *Thalassoma rueppellii* (Klunzinger, 1871) (90%, each); other common species were *Gomphosus caeruleus*, Threadfin butterflyfish, *Chaetodon auriga* Forsskål, 1775 (87%, each), Blackside hawkfish, *Paracirrhites forsteri* (Schneider, 1801 in Bloch and Schneider 1801)(84%), and *Chaetodon paucifasciatus* (81%) (Table 3).

**Saudi Arabia**

Latitude 22°

A total of 1,380 fishes were counted along the 6 transects performed in Saudi Arabia at latitude 22°N. Fish abundance ranged from 145 individuals per transect in Bostek/Thoal at 10 m deep, to 297 individuals per transect in Bostek/Thoal at 10 m deep. The mean fish abundance in all transects was 230 fish per transect. Of the 50 species considered, only 35 were reported from this latitude, with a mean of 20.7 species per transect (Fig. 3).

In terms of relative abundance (RA), the most abundant fish species was *Pseudanthias sqamipinnis* (RA= 16. 7%), followed bySohal surgeonfish *Acanthurus sohal* (Forsskål, 1775) (RA= 12.4%), *Larabicus quadrilineatus* (RA= 12.3), *Gomphosus caeruleus* (RA= 6.9), and Sunrise dottyback *Pseudochromis flavivertex* Rüppell, 1835 (RA=6.7),these five species accounted for 54.93 % of all fish species recorded from this latitude (Table 2).

In terms of frequency of appearance (FA), the most common species were Sailfin tang *Zebrasoma veliferum* (Bloch, 1795), Hooded butterflyfish *Chaetodon larvatus* Cuvier, 1831, *Heniochus intermedius* Steindachner, 1893, *Labroides dimidiatus*, *Larabicus quadrilineatus* and *Gomphosus caeruleus* (FA= 100%, each) (Table 3).

Latitude 21°

A total of 60,096 fishes were counted along the 21 transects performed in Saudi Arabia at latitude 21°. Fish abundance ranged from 116 individuals per transect in Alkabeera site at 4 m deep to 9,480 individuals per transect in South Batch Bayada at 10 m deep. The mean fish abundance in all transects was 2861.7 individuals per transect. Of the 50 species considered, only 40 were reported from this latitude, with a mean of 20.5 species per transect (Fig. 3).

In terms of relative abundance (RA), the most abundant fish species was *Chromis viridis* (RA=73.9%), followed by *Dascyllus aruanus* (RA=17.3%),and *Pseudanthias sqamipinnis* (RA= 3.9%). These three species accounted 95.04 % of all fish species recorded from this latitude (Table 2).

In terms of frequency of appearance (FA), the most common species were *Gomphosus caeruleus* and *Labroides dimidiatus*, (FA= 100%, each)followed by *Chaetodon austriacus* (FA=95.5%), Diagonal butterflyfish *Chaetodon fasciatus* Forsskål, 1775 and *Paracirrhites forsteri* (FA= 90.5%%, each) (Table 3).

Latitude 20°

A total of 51,395 fishes were counted along the 6 transects performed in Saudi Arabia at latitude 20°. Fish abundance ranged from 1278 individuals per transect in Alleeth at 10 m deep, to 20,038 individuals per transect in Alleeth at 5 m deep. The meanfish abundance of all transects was 8565.8 individuals per transect. Out of the 50 species considered, only 40 were reported from this latitude, with a mean of 21.8 species per transect (Fig. 3).

In terms of relative abundance (RA), the most abundant fish species was *Chromis viridis* (RA=92.32%), followed by *Paracheilinus octotaenia* (RA=3.86%), and *Pseudanthias squamipinnis* (RA= 1.53%). These three species accounted for 97.71 % of all fish species recorded from this latitude (Table 2).

In terms of frequency of appearance (FA), the most common species were *Acanthurus sohal*, *Chaetodon larvatus*, *Heniochus intermedius*, *Gomphosus caeruleus*, *Labroides dimidiatus*, *Larabicus quadrilineatus*, Regal angelfish *Pygoplites diacanthus* (Boddaert, 1772) and *Chromis viridis* (FA= 100%, each) (Table 3).

**Yemen**

Latitude 15°

A total of 129,932 fishes were counted during the 18 transects that were performed at latitude 15°. The mean fish abundance ranged from 95 individuals per transect at Al-Murk at 5 m deep, to 24,906 individuals per transect at Quish at 4m depth. The average fish abundance calculated from all transects was 7,218 individuals per transect. Of the 50 ornamental fish species considered, only 28 were reported from the Yemeni sites. The number of species observed ranged from 11 at Al-Murk (4 m depth) to 18 at both Quish and Al-Badi Island (shallow depth), with a mean of 11.1 species per transect (Fig. 3).

In terms of relative abundance (RA), the most abundant species was *Chromis viridis*. This species accounted for 88.5% of the fish abundance recorded at Kamaran, 79.1% at Tekfash, 93.3% at Quish and 85.3% at Al-Badi.

The mean relative abundance for *Chromis viridis* in all transects was 88.8%. The second most abundant fish species was *Dascyllus marginatus* accounting for 4.1%, 14.1%, 3.1% and 8.7% of fish abundance from Kamaran, Tekfash, Quish and Al-Badi respectively. The mean relative fish abundance for *Dascyllus marginatus* in all transects was 5.8%. This was followed by *Larabicus quadrilineatus* (average 1.6%) and *Chaetodon larvatus* (average 0.8%). These four species made up 99% of the 28 fish species which were counted (Table 2).

In terms of frequency of appearance (FA), the most common fish species were Yellowbar angelfish *Pomacanthus maculosus* (Forsskål, 1775), Moon Wrasse *Thalassoma lunare* (Linnaeus, 1758), *Larabicus quadrilineatus*, and *Chaetodon larvatus* (100%, each), followed by *Pomacanthus asfur* (Forsskål, 1775) (94.0%) and *Heniochus intermedius* (83.3%) (Table 3).

**Djibouti**

Latitude 12°

A total of 5,368 fishes were counted in the 3 transects that were examined at latitude 12°. Fish abundance ranged from 1164 fishes in Gehere at 4 m depth, to 2408 in Gehere at 4 m depth. The mean fish abundance of all transects was 1789.3 individuals per transect. Of the 50 species considered, only 18 were reported from this latitude, with a mean of 13.3 species per transect (Fig. 3).

In terms of relative abundance (RA), the most abundant fish species was *Chromis viridis* (RA=83.8%), followed by *Thalassoma lunare* (RA= 7.6%), and *Larabicus quadrilineatus* (RA= 4.1%). These three species accounted 95.51 % of all fish species recorded from this latitude (Table 2).

In terms of frequency of appearance (FA), the most common species were *Chaetodon larvatus*, Arabian butterflyfish *Chaetodon melapterus* Guichenot, 1863, Vagaband butterflyfish *Chaetodon vagabundus* Linnaeus, 1758, *Heniochus intermedius*, *Gomphosus caeruleus*, *Labroides dimidiatus*, *Larabicus quadrilineatus*, *Thalassoma lunare*, *Pomacanthus maculosus*, and *Chromis viridis* (100%, each) (Table 3).

Latitude 11°

A total of 48,488 fishes were counted along the 15 transects that were examined in Djibouti at latitude 11°. Fish abundance ranged from 181 fishes in Khor Ambado at 7 m depth, to 16,609 in Maskali at 5 m depth. The mean fish abundance of all transects was 3232.5 individuals per transect. Of the 50 species considered, only 33 were reported from this latitude, with a mean of 18.8 species per transect (Fig. 3).

In terms of relative abundance (RA), the most abundant fish species was *Chromis viridis* (RA= 90.81%), followed by *Larabicus quadrilineatus* (RA= 1.70%),and *Thalassoma lunare* (RA= 1.47%) (Table 2). These three species accounted for 93.98 % of all fish species recorded from this latitude.

In terms of frequency of appearance (FA), the most common species were Yellowtail tang *Zebrasoma xanthurum* (Blyth, 1852), *Chaetodon larvatus*, *Larabicus quadrilineatus* and *Thalassoma lunare* (100%, each), followed by *Chaetodon melapterus*, *Heniochus intermedius*, *Gomphosus caeruleus* and *Chromis viridis* (93.30%, each), see Table 3.

### All Latitudes

A total of 522,523 fish individuals were counted in the 118 transects that were examined at all latitudes (Jordan 29°, Egypt 27°, Saudi Arabia 22°, 21° and 20°, Yemen 15°, Djibouti 12° and 11°). The mean fish abundance in all transects was 4428.2 ±87.26 individuals per transect.

In terms of relative abundance (RA), the most abundant fish species was *Chromis viridis* (RA=54.4%), followed by *Pseudanthias squamipinnis* (RA= 34.7), *Dascyllus aruanus* (RA= 2.6%), *Dascyllus marginatus* (RA= 2.0), *Paracheilinus octotaenia* (RA=1.0)and *Thalassoma rueppellii* (0.7%). These six species accounted for 95.47% of all fish species recorded during this study (Table 2). Number of species, number of individuals, and Shannon-Wiener diversity in sites along the Jordanian, Egyptian, Saudi Arabia, Yemeni and Djiboutian reefs are shown in Figure 3. Of the 50 fish species counted during this study, the highest number of species (S) was found at latitude 20° on Saudi Arabian reefs (S= 21.8), and the lowest at latitude 15° in Yemen (S= 11.11). The highest mean fish abundance was found on reefs at latitude 20° in Saudi Arabia (8565.8), followed by latitude 15° in Yemen (AA=7218), whereas the lowest number of individuals was found on reefs at latitude 22° in Saudi Arabia (AA= 230). The highest mean Shannon-Wiener diversity Index was found in Saudi Arabia at latitude 22° (H`= 2.4), followed latitude 29° in Jordan, whereas the lowest was found at latitude 20° in Saudi Arabia (H`= 0.6) (Fig. 3). Table 4 shows mean fish abundance for the 50 ornamental fish species at all latitudes.

**Table 4.** Mean fish abundance in percentage (%, ± SE) per 500m² transect along reefs at JO= Jordan latitude 29°; EG=Egypt latitude 27°; SA=Saudi Arabia latitude 22, 21, 20°; YE=Yemen 15° and DJ=Djibouti latitude 12, 11°.

**Fish species**	**JO 29°**	**EG 27°**	**SA 22°**	**SA 21°**	**SA 20°**	**YE 15°**	**DJ 12°**	**DJ 11°**
**Scorpaenidae**								
*Pterois miles*	0.2 ±0.1	0.6 ±0.2	0.0 ±0.0	0.0 ±0.0	0.0 ±0.0	0.0 ±0.0	0.0 ±0.0	0.0 ±0.0
*Pterois radiata*	0.4 ±0.2	0.5 ±0.1	0.0 ±0.0	0.0 ±0.0	0.2 ±0.2	0.0 ±0.0	0.0 ±0.0	0.3 ±0.2
**Serranidae**								
*Pseudanthias squamipinnis*	666.2 ±156.2	5356.2 ±896.0	38.3 ±21.2	111.4 ±51.4	130.8 ±59.6	0.0 ±0.0	0.0 ±0.0	0.0 ±0.0
**Pseudochromidae**								
*Pseudochromis flavivertex*	0.0 ±0.0	0.0 ±0.0	15.3 ±7.1	0.5 ±0.3	0.0 ±0.0	3.3 ±1.8	0.0 ±0.0	0.0 ±0.0
*Pseudochromis fridmani*	13.3 ±2.9	26.1 ±8.3	2.0 ±0.9	4.7 ±1.5	7.8 ±6.5	0.0 ±0.0	0.0 ±0.0	0.0 ±0.0
*Pseudochromis springeri*	2.3 ±0.6	2.5 ±1.8	0.0 ±0.0	0.0 ±0.0	0.0 ±0.0	0.0 ±0.0	0.0 ±0.0	0.0 ±0.0
**Chaetodontidae**								
*Chaetodon auriga*	0.2±0.1	13.6 ±4.9	2.8 ±0.7	3.0 ±0.8	0.7 ±0.4	0.0 ±0.0	0.0 ±0.0	0.0 ±0.0
*Chaetodon austriacus*	8.2 ±1.6	10.7 ±1.5	5.3 ±1.3	6.5 ±0.8	2.2 ±1.4	0.0 ±0.0	0.0 ±0.0	0.0 ±0.0
*Chaetodon fasciatus*	2.2 ±0.6	3.6 ±0.7	3.0 ±1.3	3.1 ±0.5	0.8 ±0.4	0.4 ±0.3	0.7 ±0.7	2.7 ±0.7
*Chaetodon larvatus*	0.0 ±0.0	0.1 ±0.1	2.7 ±0.7	1.9 ±0.4	17.5 ±4.3	58.1 ±12.2	3.3 ±0.7	13.3 ±2.6
*Chaetodon lineolatus*	0.0 ±0.0	0.2 ±0.1	0.3 ±0.3	0.1 ±0.1	0.0 ±0.0	0.0 ±0.0	0.0 ±0.0	0.0 ±0.0
*Chaetodon melannotus*	0.6 ±0.2	3.1 ±0.9	0.0 ±0.0	2.2 ±1.4	0.0 ±0.0	0.0 ±0.0	0.0 ±0.0	0.3 ±0.2
*Chaetodon melapterus*	0.0 ±0.0	0.0 ±0.0	0.0 ±0.0	0.0 ±0.0	0.0 ±0.0	0.0 ±0.0	7.3 ±1.2	7.1 ±1.3
*Chaetodon mesoleucos*	0.0 ±0.0	0.0 ±0.0	0.0 ±0.0	0.6 ±0.2	0.3 ±0.3	0.9 ±0.6	0.0 ±0.0	1.7 ±0.4
*Chaetodon paucifasciatus*	24.5 ±2.3	7.2 ±1.2	0.0 ±0.0	2.3 ±0.6	0.3 ±0.3	0.0 ±0.0	0.0 ±0.0	0.0 ±0.0
*Chaetodon semilarvatus*	0.0 ±0.0	1.5 ±0.4	0.2 ±0.2	0.7 ±0.2	1.8 ±0.9	2.1 ±0.8	1.3 ±1.3	2.5 ±0.6
*Chaetodon trifascialis*	0.2 ±0.1	9.2 ±1.5	0.0 ±0.0	1.0 ±0.3	0.0 ±0.0	0.6 ±0.6	1.3 ±1.3	2.2 ±1.0
*Chaetodon vagabundus*	0.0 ±0.0	0.0 ±0.0	0.0 ±0.0	0.0 ±0.0	0.0 ±0.0	0.0 ±0.0	6.0 ±1.2	2.3 ±0.6
*Heniochus acuminatus*	0.0 ±0.0	0.0 ±0.0	0.0 ±0.0	0.0 ±0.0	0.0 ±0.0	0.0 ±0.0	0.0 ±0.0	0.1 ±0.1
*Heniochus intermedius*	1.1 ±0.3	2.8 ±0.4	7.8 ±2.00	2.6 ±0.4	3.5 ±0.6	8.2 ±2.1	2.7 ±0.7	9.6 ±3.9
**Pomacanthidae**								
*Pomacanthus asfur*	0.0 ±0.0	0.0 ±0.0	3.8 ±1.5	0.0 ±0.0	3.7 ±1.1	9.8 ±1.8	1.7 ±1.7	4.2 ±0.7
*Pomacanthus imperator*	0.1 ±0.1	0.3 ±0.1	0.0 ±0.0	0.0 ±0.0	0.2 ±0.2	0.1 ±0.1	0.3 ±0.3	0.0 ±0.0
*Pomacanthus maculosus*	0.0 ±0.0	0.4 ±0.1	1.2 ±0.5	0.4 ±0.4	1.7 ±0.5	20.2 ±3.9	2.0 ±0.6	2.2 ±0.6
*Pygoplites diacanthus*	0.2 ±0.2	1.3 ±0.3	1.5 ±0.8	4.3 ±0.8	2.3 ±0.3	0.0 ±0.0	0.0 ±0.0	4.1 ±0.9
**Pomacentridae**								
*Amphiprion bicinctus*	25.2 ±2.0	2.8 ±1.4	0.8 ±0.5	1.2 ±0.3	1.7 ±0.6	0.1 ±0.1	0.0 ±0.0	1.3 ±0.7
*Chromis viridis*	450.0 ±158.6	657.6 ±209.2	0.0 ±0.0	2114.3 ±582.7	7908.3 ±3192.2	6411.1 ±2248.9	1500.0 ±346.4	2935.3 ±1093.6
*Dascyllus aruanus*	125.7 ±32.7	10.7 ±5.00	1.3 ±0.9	494.o ±185.3	10.3 ±7.8	24.0 ±17.8	0.0 ±0.0	12.53 ±6.2
*Dascyllus marginatus*	111.8 ±24.2	17.4 ±7.8	0.0 ±0.0	0.0 ±0.0	5.8 ±4.2	420.9 ±124.2	0.0 ±0.0	2.7 ±1.5
*Dascyllus trimaculatus*	7.2 ±2.9	0.8 ±0.4	0.0 ±0.0	1.7 ±1.2	9.5 ±7.1	72.9 ±72.8	0.0 ±0.0	1.3 ±1.1
**Cirrhitidae**								
*Paracirrhites forsteri*	0.0 ±0.0	6.4 ±1.1	0.7 ±0.4	9.5 ±1.7	0.2 ±0.7	0.0 ±0.0	0.0 ±0.0	0.0 ±0.0
**Labridae**								
*Anampses twistii*	9.8 ±1.7	7.0 ±1.1	1.2 ±0.8	2.7 ±0.6	1.2 ±0.7	0.0 ±0.0	0.0 ±0.0	1.0 ±0.3
*Bodianus anthioides*	2.0 ±0.6	1.6 ±0.6	1.0 ±0.5	0.2 ±0.1	0.8 ±0.8	0.0 ±0.0	0.0 ±0.0	0.0 ±0.0
*Cheilinus lunulatus*	0.3 ±0.2	2.1 ±0.4	3.0 ±1.1	0.4 ±0.3	1.5 ±0.6	3.5 ±0.1	0.3 ±0.3	1.6 ±0.9
*Coris aygula*	0.3 ±0.1	4.7 ±1.1	0.3 ±0.3	0.2 ±0.1	0.0 ±0.0	0.1 ±0.1	0.0 ±0.0	0.0 ±0.0
*Gomphosus caeruleus*	8.3 ±1.6	13.1 ±2.1	15.8 ±1.7	15.8 ±1.9	14.3 ±3.7	0.4 ±0.2	21.0 ±10.6	15.7 ±3.5
*Labroides dimidiatus*	1.7 ±0.5	11.0 ±1.5	8.3 ±1.6	6.4 ±0.7	4. 0 ±0.7	0.4 ±0.2	3.0 ±1.0	2.9 ±0.9
*Larabicus quadrilineatus*	6.8 ±1.1	3.9 ±0.7	28.3 ±7.2	6.0 ±1.7	34.8 ±4.5	114.6 ±21.7	73.7 ±25.7	55.1 ±6 .0
*Novaculichthys taeniourus*	0.00 ±0.00	0.00 ±0.00	0.00 ±0.00	0.90 ±0.90	0.17 ±0.17	0.00 ±0.00	0.00 ±0.00	0.00 ±0.00
*Paracheilinus octotaenia*	119.9 ±44.8	17.1 ±9.6	2.5 ±1.7	0.0 ±0.0	330.8 ±144.00	5.6 ±5.6	0.0 ±0.0	21.1 ±9.7
*Thalassoma rueppellii*	42.0 ±4.4	78.7 ±23.5	14.0 ±3.7	19.8 ±3.9	13.7 ±5.9	0.0 ±0.0	0.0 ±0.0	0.0 ±0.0
*Thalassoma lunare*	0.8 ±0.4	2.6 ±0.7	12.8 ±3.2	6.3 ±1.3	17.7 ±7.7	44.4 ±8.2	135.3 ±5.4	47.6 ±8.4
**Acanthuridae**								
*Acanthurus sohal*	0.0 ±0.0	41.4 ±21.7	28.5 ±13.5	8.9 ±1.8	26.5 ±8.3	10.8 ±5.3	8.0 ±4.2	15.0 ±5.8
*Naso lituratus*	0.1 ±0.1	4.2 ±1.7	6.7 ±4.1	4.1 ±0.9	4.3 ±2.4	1.4 ±1.4	0.0 ±0.0	27.3 ±27.3
*Zebrasoma veliferum*	1.1 ±0.3	4.9 ±1.5	14.8 ±8.3	13.00 ±9.5	4.2 ±1.9	3.1 ±1.1	0.0 ±0.0	6.6 ±1.3
*Zebrasoma xanthurum*	4.1 ±0.5	3.7 ±1.5	2.0 ±1.6	6.3 ±4.0	0.3 ±0.3	1.3 ±0.7	21.3 ±13.7	32.1 ±6.4
**Balistidae**								
*Balistapus undulatus*	0.8 ±0.3	0.9 ±0.3	1.8 ±0.7	2.9 ±0.6	1.2 ±0.7	0.0 ±0.0	0.0 ±0.0	0.7 ±0.3
*Balistoides viridescens*	0.0 ±0.0	0.0 ±0.0	0.3 ±0.2	0.1 ±0.1	0.3 ±0.3	0.0 ±0.0	0.0 ±0.0	0.1 ±0.1
*Rhinecanthus assasi*	0.0 ±0.0	0.5 ±0.2	0.0 ±0.0	1.4 ±0.4	0.2 ±0.2	0.1 ±0.1	0.0 ±0.0	0.00 ±0.00
**Ostraciidae**								
*OstraciOstracion cubicus*	0.5 ±0.3	0.0 ±0.0	0.5 ±0.5	0.1 ±0.1	0.0 ±0.0	0.1 ±0.1	0.0 ±0.0	0.00 ±0.00
**Tetraodontidae**								
*Arothron diadematus*	0.1 ±0.1	2.1 ±1.3	0.8 ±0.3	0.2 ±0.1	0.2 ±0.2	0.0 ±0.0	0.0 ±0.0	0.00 ±0.00

## Fish families

**Figure 4.**
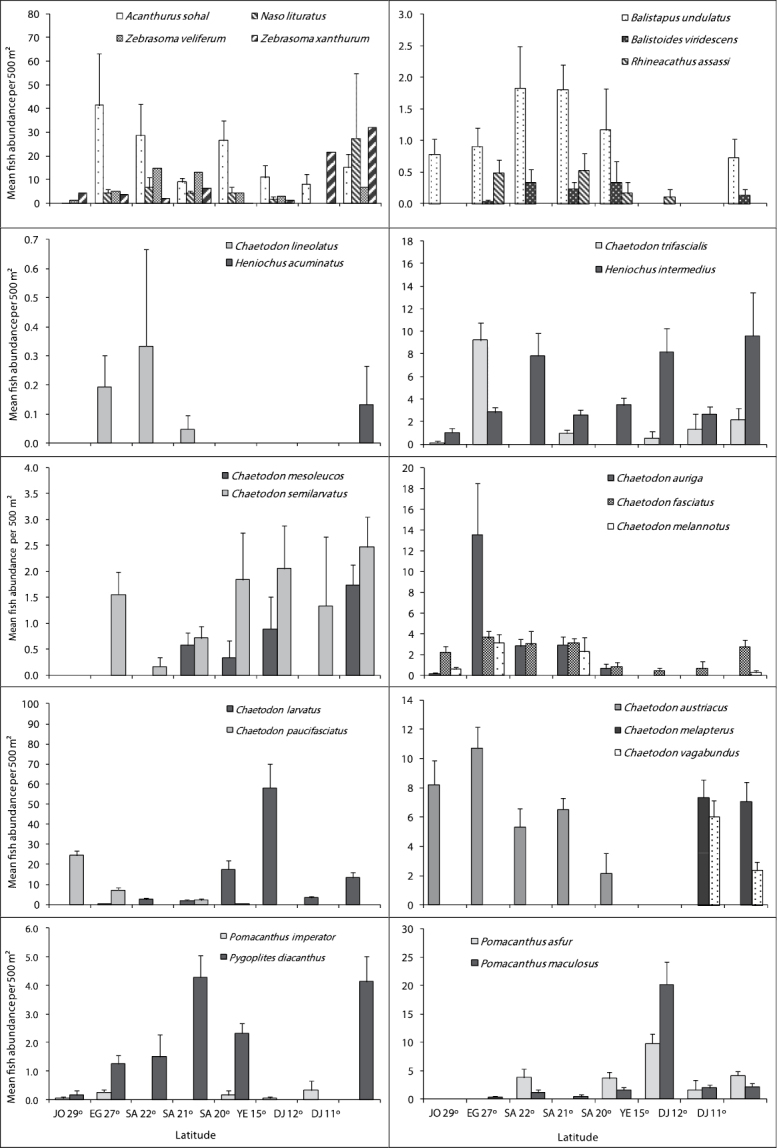
Differences in mean fish abundance per 500 m² transect according to latitudinal distribution for the families: Acanthuridae (*Acanthurus sohal*, *Naso lituratus*, *Zebrasoma veliferum* and *Zebrasoma xanthurum*), Balistidae (*Balistoides undulates*, *Balistoides viridescens*, and *Rhinecanthus assasi*), Chaetodontidae (*Chaetodon lineolatus*, *Heniochus acuminatus*, *Chaetodon mesoleucos*, *Chaetodon semilarvatus*, *Chaetodon auriga*, *Chaetodon fasciatus*, *Chaetodon melannotus*, *Chaetodon larvatus*, *Chaetodon paucifasciatus*, *Chaetodon austiacus*, *Chaetodon trifascialis*, *Heniochus intermedius*, *Chaetodon melapterus*, and *Chaetodon vagabundus*), and Pomacanthidae (*Pomacanthus asfur*, *Pomacanthus imperator*, *Pomacanthus maculosus* and *Pygoplites diacanthus*).

**Figure 5.**
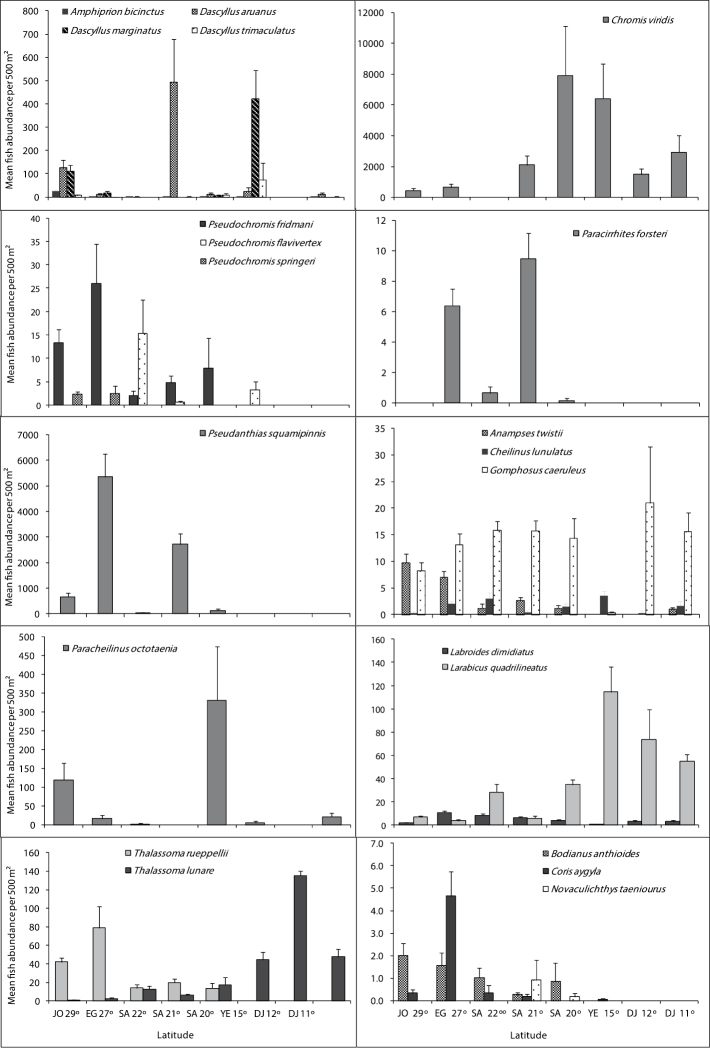
Differences in mean fish abundance per 500 m² transect according to latitudinal distribution for the fish species belonging mainly to families: Pomacentridae (*Amphiprion bicinctus*, *Dascyllus aruanus*, *Dascyllus marginatus*, *Dascyllus trimaculatus*, and *Chromis viridis*), Pseudochromidae (*Pseudochromis fridmani*, *Pseudochromis flavivertex* and *Pseudochromis springeri*), Cirrhitidae (*Paracirrhites forsteri*), Serranidae (*Pseudanthias squamipinnis*), and Labridae (*Anampses twistii*, *Cheilinus lunulatus*, *Gomphosus caeruleus*, *Paracheilinus octotaenia*, *Labroides dimidiatus*, *Larabicus qudrilineatus*, *Thalassoma rueppellii*, *Thalassoma lunare*, *Bodianus anthioides*, *Coris aygula* and *Novaculichthys taeniourus*).

**Figure 6.**
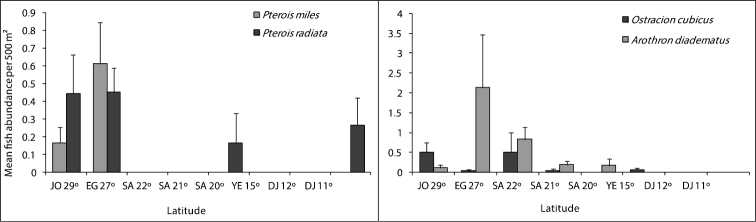
Differences in mean fish abundance per 500 m² transect according to latitudinal distribution for the fish species belonging to the family Scorpaeinidae (*Pterois miles*, *Pterois radiata*), Ostraciidae (*Ostracion cubicus*), and Tetraodontidae (*Arothron diadematus*).

Figures 4, 5 and 6 illustrate differences in the mean fish abundance per 500 m² transect, according to latitudinal distribution among the 50 ornamental fish species. These belong mainly to the following fish families: [Fig. 4. I. Acanthuridae (4 species), II. Balistidae (3 species), III. Chaetodontidae (14 species), IV. Pomacanthidae (4 species)[; [Fig. 5. V. Pomacentridae (5 species), VI. Pseudochromidae (3 species), VII. Cirrhitidae (one species), VIII. Serranidae (one species) IX. Labridae (11 species)]; and Fig. 6. X. Scorpaenidae (2 species), XI. Ostraciidae (one species), XII. Tetraodontidae [(one species)] that are utilized by the aquarium trade in the region.

**Acanthuridae (Surgeonfishes)**

Of the 12 species of surgeonfish’s reported from the Red Sea, only 4 speciesare important in the ornamental fish trade, therefore only *Acanthurus sohal*, Orangespine unicornfish *Naso lituratus* (Forster, 1801 in Bloch and Schneider 1801), *Zebrasoma veliferum* and *Zebrasoma xanthurum* were counted. *Acanthurus sohal* were abundant along Egyptian latitude 27° followed by Saudi Arabian latitude 22°. *Naso lituratus* were abundant along the Djiboutian reefs at latitude 11° (Fig 4). *Zebrasoma veliferum* were abundant along the Saudi Arabian reefs at latitudes 22 and 21°. Whereas, *Zebrasoma xanthurum* were most abundant along the Djiboutian reefs at latitudes 11 and 12°. Triggerfishes had the lowest number of individuals.

**Balistidae (Triggerfishes)**

This family had the lowest number of individuals of all fish families examined here. Out of the 10 triggerfishes reported from the Red Sea, only Ornage-lined triggerfish *Balistapus undulatus* (Park, 1797), Titan triggerfish *Balistoides viridescens* (Bloch & Schneider, 1801), and Picasso triggerfish *Rhinecanthus assasi* (Forsskål, 1775) were counted due to their importance in the aquarium trade. Geographical trends indicated that *Balistoides undulates* and *Balistoides viridescens* were more abundant along the Saudi Arabian reefs at Latiudes 22, 21 and 20° but *Rhinecanthus assasi* did not show any clear geographical trend (Fig. 4).

**Chaetodontidae (Butterflyfishes)**

Out of the 17 butterflyfishes known from the Red Sea, this study indicates the presence of 14 species. Geographical trends were observed in almost all butterflyfish species (Fig. 4). *Chaetodon austiacus* and *Chaetodon paucifasciatus* were most abundant along the reefs at latitudes 29 and 27°. These two species were not recorded in latitudes 15, 12 and 11°. On the other hand, *Chaetodon auriga*, *Chaetodon fasciatus* and *Chaetodon lineolatus* Cuvier, 1831 in Cuvier and Valenciennes were most abundant along the Egyptian and Saudi Arabia at latitudes 27, 22, and 21°. *Chaetodon larvatus* was most abundant along the Saudi Arabia and Yemen reefs at latitudes 20 and 15°. There were 3 butterflyfish species *Chaetodon melapterus*, *Chaetodon vagabundus*, and Pennant coralfish *Heniochus acuminatus* (Linnaeus, 1758) exist only at the Djiboutian reefs at latitudes 12 and 11°. The distributional pattern for all butterflyfish species is shown in Fig. 4.

**Pomacanthidae (Angelfishes)**

This family had the second lowest number of individuals of the fish families examined here. Out of the 7 species of angelfish recorded from the Red Sea, only 4 species (*Pomacanthus asfur*, Emperor angelfish, *Pomacanthus imperator* (Bloch, 1787), *Pomacanthus maculosus*, and *Pygoplites diacanthus*) that are used in the aquarium fish trade in the Red Sea were found. Latitude 15° exhibited the highest number of individuals for angelfish species, particularly for *Pomacanthus asfur* and *Pomacanthus maculosus*. Whereas, the reefs at the latitude 29° had the lowest number of individuals. Geographical trends were clear for some species like *Pomacanthus asfur* which is the most abundant angelfish at the latitudes 15° but did not reach the northern Red Sea at the reefs between latitudes 29 and 27°.The distributional pattern for angelfish species is shown in Fig. 4.

**Psudochromidae (Dottybacks)**

Out of the 12 dottybacks fishes known from the Red Sea, only 3 species of importance in ornamental fish trade were counted. The 3 of them were not reported along the Djiboutian reefs. Bluestriped dottyback *Pseudochromis springeri* Lubbock, 1975 preferred the north, and it was found only along the Jordanian and Egyptian reefs at latitudes 29 and 27°. Whereas, Orchid dottyback *Pseudochromis fridmani* Klausewitz, 1968 extended to reach reefs at latitude 15°. The distributional pattern for Dottybacks is shown in Fig. 4.

**Pomacentridae (Damselfishes)**

Damselfishes had the highest number of individuals at all studied sites. Out of 35 damselfishes known from the Red Sea, only 5 species that are mostly used in the aquarium trade in the region were counted. *Chromis viridis* was the most dominant species of the damselfish family on Red Sea reefs. Geographical distribution of the clownfish *Amphiprion bicinctus* revealed that it prefers the Jordanian and Egyptian reefs at latitude 29° and 27°. The distributional pattern for damselfishes is shown in Fig. 5.

**Labridae (Wrasses)**

Out of the 64 wrasses reported from the Red Sea, only 11 species that are used mostly in the aquarium fish trade were counted. *Thalassoma rueppellii*, *Bodianus anthioides* (Bennett, 1832), *Coris aygula* Lacepède, 1801 and *Anampses twistii* were most abundant along the reefs in latitudes 29 and 27°; they were rare or not recorded along the Yemeni and Djiboutian reefs at latitudes 15, 12 and 11°. However, *Thalassoma rueppellii* and *Bodianus anthioides* were restricted to the northern and central Red Sea. On the other hand, *Thalassoma lunare* was most abundant in the south at latitudes 15, 12 and 11°.The cleaner wrasse *Labroides dimidiatus* wasmost abundant along the reefs in latitudes 27, 22° and *Larabicus qudrilineatus* on the other side was most abundant along the reefs at latitudes 15, 12 and 11°. The distributional pattern for wrasses is presented in Fig. 5.

**Serranidae (Groupers)**

Only *Pseudanthias squampinnis* belonging to the subfamily Anthininae was counted during this study. The counts indicated that this species was most abundant along the reefs at latitudes 27 and 29° respectively and it was not reported from the Yemeni and Djiboutian reefs at latitudes 15, 12 and 11° (Fig. 5).

**Cirrhitidae (Hawkfishes)**

*Paracirrhites forsteri* did not show a geographical trend in its distribution, except that it was not recorded in reefs at latitudes 29°, 15, 12 and 11° (Fig. 5).

**Ostracidae (Boxfishes)**

The distributional patterns for the boxfish, *OstraciOstracion cubicus* Linnaeus, 1758, are shown in Fig. 6. It was abundant along the Jordanian reefs at latitude 29° and Saudi Arabia 22° reefs. It was either rare or not reported in other studied sites.

**Tetraodontidae (Pufferfishes)**

*Arothron diadematus* (Rüppell, 1829) is of importance in the fish aquarium trade in the region, and the number of individuals were very low. This species was not reported in the southern Red Sea and Gulf of Aden (Fig. 6).

**Scorpaenidae (Scorpionfishes)**

Two species *Pterois miles* (Bennett, 1828) and *Pterois radiata* Cuvier, 1829 in Cuvier and Valenciennes 1829 belonging to this family were counted; *Pterois miles* was reported only on Jordanian and Egyptian reefs. Whereas, *Pterois radiata* reached to Djiboutian waters, but both species were present in very low numbers. The distributional pattern for both species is shown in Fig. 6.

### Correlation of fish community pattern with benthic habitat

The simple regression procedure of the Stat View software was used to correlate the fish community patterns such as species richness, average fish abundance (N), and Shannon-Wiener Index (H`) to benthic habitat (Table 5). The maximum regression correlation between hard coral cover and fish species richness (r=0.9060) was at reefs in latitude 12° followed by latitude 22°. Whereas, the strongest correlation with average fish abundance (r=0.554) was at reefs in latitude 20°, followed by latitude 15°. Reefs in latitude 20° also exhibited the highest regression correlation with Shannon-Wiener diversity (r=0.7582), followed by latitude 15° (Table 5). However, the highest correlation between soft coral and species richness was (r=0.3420) at latitude 21°, followed by latitude 22° (Table 6). Whereas, the maximum regression correlation with average fish abundance was found (r=0.9838) at latitude 12°, followed by latitude 21°. Reefs at Latitude 12° exhibited the highest regression correlation with Shannon-Wiener diversity (r=0.9869), followed by latitude 20°.

**Table 5.** Regression correlation (r) analysis of fish community pattern vs. hard coral (simple regression); mean fish abundance (N), species richness (number of species) and Shannon-Wiener Index (H`) at reefs at various latitudes along the Red Sea and Gulf of Aden * if *p* <0.05 significant correlation.<br/>

**Latitude**	**Number of species**	**Number of Individuals**	**Shannon-Wiener diversity H´**
**29°**	**0,257**	**0,378**	**0,219**
	*p*=0.305	*p=0.121*	p=0.385
**27**°	**0,395**	**0,354**	**0,451**
	*p*=0.028*	*p*=0.051	*p*=0.01*
**22**°	**0,179**	**0,473**	**0,118**
	*p*=0.733	*p*=0.343	*p*=0.807
**21**°	**0,415**	**0,447**	**0,2**
	*p*=0.613	*p*=0.042*	*p*=0.385
**20**°	**0,767**	**0,554**	**0,758**
	*p*=0.075	*p*=0.254	*p*=0.080
**15**°	**0,663**	**0,221**	**0,584**
	*p*=0.003*	*p*=0.375	*p*=0.011
**12**°	**0,906**	**0,431**	**0,448**
	*p*=0.278	*p*=0.716	*p*=0.711
**11**°	**0,02**	**0,305**	**0,071**
	*p*=0.944	*p*=0.268	*p*=0.793

**Table 6.** Regression correlation (r) analysis of fish community pattern vs. soft coral (simple regression); mean fish abundance (N), species richness (number of species) and Shannon-Wiener Index (H`) at reefs at various latitudes along the Red Sea and Gulf of Aden. * if *p* <0.05 significant correlation.

**Latitude**	**Number of species**	**Number of Individuals**	**Shannon-Wiener diversity H´**
**29°**	**0,329**	**0,311**	**0,445**
	*p=*0.183	*p=*0.209	*p*=0.064
**27°**	**0,122**	**0,355**	**0,564**
	*p=*0.509	*p*=0.050*	*p=*0.001*
**22°**	**0,338**	**0,276**	**0,484**
	*p*=0.513	*p*=0.598	*p*=0.330
**21°**	**0,342**	**0,475**	**0,422**
	*p*=0.128	*p*=0.030	*p*=0.0.056
**20°**	**0,237**	**0,095**	**0,758**
	*p*=0.650	*p*=0.862	*p*=0.897
**15°**	**0**	**0**	**0**
	*p*=1.000	*p*=1.000	*p*=1.000
**12°**	**0,19**	**0,984**	**0,99**
	*p*=0.880	*p*=0.115	*p*=0.120
**11°**	**0,336**	**0,114**	**0,33**
	*p*=0.220	*p*=0.685	*p*=0.229

## Biogeography

**Figure 7.**
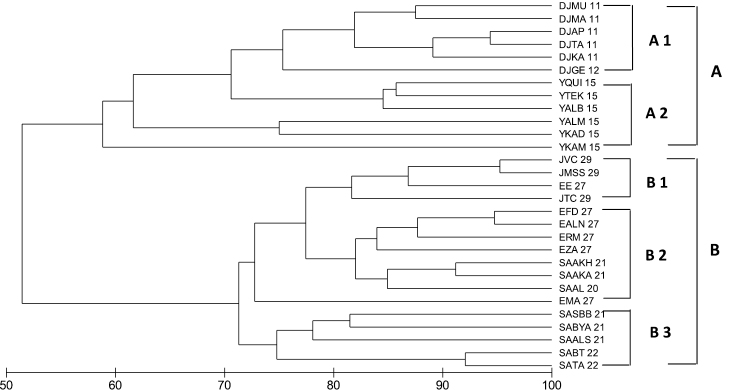
Cluster analysis of relationships between ornamental fish assemblages (Bray-Curtis similarity) from different countries in the Red Sea and Gulf of Aden region. Country Key: J29°= sites at latitude at 29° on the Jordanian coast, JMSS 29= Marine Science Station, JTC 29= Tourist Camp, JVC= Visitor Center; E27°= sites at latitude 27° in Egypt, EALN 27= AL-Noksh, EMA 27= Mahmoudat, EFD 27= Fanar Dolphin, EE 27= Elli, ERM 27= Ras Mohamad, EZA 27= Zorab; SA22, SA21 and SA20° = sites at latitudes on the Saudi Arabia coast, SATA 22= Thoal- Awjam, SABT 22= Bostek/Thoal, SAAKA 21= Alkabeera, SABYA 21= Bayada, SASBB 21= South Batch Bayada, SAALS 21= Al-Sagheera, SAAKH 21= Al-Kherq, SAAL 20= Alleeth; Y15°= sites at latitude along the Yemeni coast, YKAD 15= Kadaman, YKAM 15= Kamaran, YTEK 15= Tekfash, YQUI 15= Quish, YALM 15= Al-murk, YALB 15= Al-Badi; Dj11 and Dj12° =sites at latitudes 11 and 12° along the Djiboutian coast, DJGE 12= Gehere, DJKA 11= Khor Ambado, DJMA 11= Maskali, DJMU 11= Musha, DJTA 11= Tajoura, DJAP 11= ArtaPlaga.

Cluster analysis revealed that two primary groups of sites can be distinguished from the data (Fig. 7): Group A is divided into two sub-groups gathering the sites from Djibouti and Yemen with about 58% similarity: sub-group (A1) incorporates sites in Djibouti latitudes 12 and 11° with about 70% similarity (A2) incorporates sites in the country of Yemen (latitude 15°) with about 63% similarity, and sub-group. Group (B) is divided into 3 sub-groups gathering the sites in Jordan, Egypt and Saudi Arabiawith 71% similarity: subgroup (B1) incorporates the sites in Jordan latitude (29°) with about 82% similarity, subgroup (B2) incorporates most of the sites in Egypt at latitudes 27 and some sites in Saudi Arabia at latitudes (20, and 21°) with about 82% similarity.,and subgroup (B3) incorporates the sites is Saudi Arabia at latitudes (21 and 22°) with about 75% similarity. These two main groups (A and B) are connected together with about 52% similarity. The data clearly show that ornamental fish communities in the Red Sea and Gulf of Aden fall into two distinct biogeographical groups. One of the groups characterizes the northern and central Red Sea, whilst the other is in the southern Red Sea and Gulf of Aden.

## Discussion

A new and updated checklist of fishes of the Red Sea has recorded a total of 1078 species belonging to 154 families (Golani and Bogorodsky 2010). From this list, a total of 50 fish species used for aquarium trade were investigated during the present study. A database for aquarium fish stocks was established during this intensive survey, and is housed at PERSGA office Jeddah, Saudi Arabia.

This study revealed marked differences in the structure of ornamental fish assemblages with latitude. The presence-absence data for the fifty ornamental fish species used in the aquarium trade in the region support the presence of two main biogeographic gradients in the Red Sea and Gulf of Aden: a south Red Sea and Gulf of Aden latitudinal gradient, and a gradient along the north and central Red Sea. Latitudinal gradients in water quality (temperature, salinity, plankton production) may cause in part this north-south variation in fish community structure. There are marked changes in the structure of coral reef fish communities moving from north to the south within the Red Sea. Carter and Prince (1981) concluded that gradual changes in salinity and temperature could generate abrupt boundaries for species distributions. Differences in environmental tolerance among species could mean that some are better adapted to conditions prevailing in the south than those further north or vice-versa. Factors in the physical environment including irradiance, salinity, temperature, pressure, nutrients and wastes all influence the distribution of organisms in the sea. The ability of an organism to tolerate changes in these physical environmental factors plays a major role in determining the organism’s distribution in the marine environment (Karlenskint et al. 2008). There are strong gradients from north to south in surface water temperature (approx. 6-8 °C), salinity (5–7 ppt), nutrient concentration and turbidity in the Red Sea (Gordeyeva 1970, Morcos 1970, Morley 1975, Edwards and Head 1987, Weikert 1987).

The gradient observed during this study could also be due to different types of habitat available in the different sites, as well as their different amounts of coral cover, which could produce more or less favorable conditions for the development of juvenile fishes (availability of food, food supply, substrate complexity with different coral cover, etc.). This study identified major differences in the faunal composition and relative abundance in almost all of the fish families investigated. They included the studied species of triggerfishes (Balistidae), surgeonfishes (Acanthuridae), butterflyfishes (Chaetodontidae), angelfishes (Pomacanthidae), damselfishes (Pomacentridae), Wrasses (Labridae), dottybacks (Pseudochromidae) and *Pseudanthias squamipinnis* in the family grouperfishes (Serranidae). Similar results were obtained by (Ormond and Edwards 1987; Roberts and Ormond 1987; Roberts et al. 1992). Sheppard et al. (1992) indicated that there are marked differences among the different regions of the Red Sea in fish species richness, assemblage composition and species abundances. Brokovich et al. (2006) reported a strong correlation between fish assemblages and the different types of habitats at the northern tip of the Gulf of Aqaba. Similar results were also reported by Khalaf and Kochzius (2002).

The fish fauna of the Djiboutian coast is shared with the Indian Ocean and the Red Sea. However, in terms of species composition, the Red Sea influence dominates, especially in areas near to Bab el Mandeb (Barrat and Medeley, 1990). A biogeographic analysis of the Indian Ocean coral fauna based on presence/absence of species revealed a clear pattern of faunal relationships between the Red Sea, Southern Arabia and the Indian Ocean (Khalaf and Kochzius 2002). Habitat strongly influences which species are able to live in a particular place. There are considerable differences in reef structure and coral assemblages from north to south within the Red Sea. In the south, reef structures becomes shallow with macroalgal dominated frameworks (Yemen and Djibouti). In the far south there are few areas of hard substrata, and these are mainly coralline-algal reefs covered with dense growth of the brown algae Sargassum and Turbinaria (Khalaf, personal observation).

A review of the literature describing fish habitat correlations from various regions presents a convincing positive relationship between habitat structural complexity and reef fish diversity in the Caribbean (Risk 1972, Luckhurst and Luckhurst 1978) and in the Great Barrier Reef (McCormick 1994). The strength of this correlation however, may vary among reef types. Percent of live branching or massive coral, substratum diversity and complexity have several times been identified as important predictors of the diversity of reef fish assemblages (Talbot 1965, Talbot and Goldman 1972, Luckhurst and Luckhurst 1978, Bouchon-Navaro and Bouchon 1989). This study supports the above mentioned studies, and shows that a number of fish species occupy different latitudes. Species varied in their abundances from reef to reef, and adjacent reefs supported different groups of species. As a general conclusion, we suggest that differences among reefs and habitats were among important components of variability in the number of fishes and species of ornamental fishes along the Red Sea and Gulf of Aden.

The high numbers of the clownfish *Amphiprion bicinctus* along the Jordanian reefs at latitude 29° compared to other reefs in other latitudes is perhaps due the availability of the sea anemone hosts *Entacmaea quadricolor* and *Heteractis crispa* (Huebner et al. 2012). Chadwick and Arvedlund (2005) proposed that *Heteractis crispa* may serve as nursery habitats for *Amphiprion bicinctus* because they host more juvenile fish than does *Entacmaea quadricolor*, and Huebner et al. (2012) further documented this relationship using field experiments. The present study revealed that *Amphiprion bicinctus*, which is an endemic species to the Red Sea and Gulf of Aden, was a common and abundant species in the northern Red Sea, less common in the central Red Sea and rare or not present in the southern Red Sea and Gulf of Aden. These patterns may be due in part to the distributional and abundance patterns of the above host anemones, but detailed surveys of these anemones need to be conducted throughout the Red Sea to further test these ideas.

Two of the 3 species that belong to the family of dottybacks, Pseudochromidae i.e., *Pseudochromis springeri* and *Pseudochromis fridmani* were endemic to the Red Sea (Randall 1992), and both of them were not found along the Yemeni and Djiboutian reefs. The third species *Pseudochromis flavivertex* which is endemic to the Red Sea and Gulf of Aden (Randall 1992) was recorded in all latitudes except at latitudes 12 and 11° along the Djiboutian reefs during this investigation. A total of 14 butterflyfishes are reported from the Red Sea, of which seven are endemic or range no further than the Gulf of Aden (Randall, 1992). In the present study, 14 species of butterflyfishes have also been reported. Butterflyfish assemblages in the southern Red Sea differ from that in the north (Righton et al. 1996). Other differences occur among butterflyfish. For example, *Chaetodon paucifasciatus* is abundant in the Gulf of Aqaba and Gulf of Suez but absent or rare in the southern Red Sea. However, *Chaetodon larvatus* is a dominant species in the south but rare in the northern Red Sea and Gulf of Suez and absent entirely from the Gulf of Aqaba. On the other hand, there are some species such as *Heniochus acuminatus*, *Chaetodon melapterus* and *Chaetodon vagabundus* that are present along the Djiboutian reefs but are not reported in the Red Sea. The results presented in this study demonstrate that there are many differences between the reef fish fauna of the northern and central Red Sea, from that of the southern Red Sea and Gulf of Aden. For example, *Pseudanthias squamipinnis* dominated fish assemblages in the northern Red Sea along the Jordanian and Egyptian coasts, whereas *Chromis viridis* dominated fish assemblages in the central Red Sea, along the Saudi Arabia coast at both latitudes 20 and 21°, and in the Gulf of Aden.

During this survey, *Chromis viridis* was the most abundant species in the Red Sea and Gulf of Aden. This species dominated the reefs at latitudes 21, 20, 15, 12 and 11°. Similar results were reported at Nuweiba (Ben-Tuvia, 1983), at Djiboutian reefs (Barrat and Medley 1990), at Sanganeb atoll (Krupp et al. 1993), and in Eritrean reefs (Daw et al. 2001). *Chromis viridis* usually forms large aggregations and is found associated with large heads of *Acropora* corals that provide shelter from predators and a nocturnal retreat. Thus, these distributional patterns for this fish may indicate a higher abundance of large *Acropora* corals on southern than northern reefs in this region. The second most abundant species was *Pseudanthias squamipinnis*, which dominates the reefs at latitudes 29, 27 and 22°. Similar findings were recorded at the Japanese Garden reef site at Eilat (Rilov and Benayahu 2000), at Nuweiba (Ben-Tuvia et al. 1983), at Sanganeb Atoll (Krupp et al. 1993), and on Jordanian reefs (Khalaf and Kochzius 2002). This species occurs usually in small to very large aggregations around rock or coral heads. These two species feed on plankton above rich beds of live coral (Khalaf and Disi 1997). These patterns indicate the importance of live coral cover, reef rugosity, and availability of plankton in the distribution of these reef fishes.
